# Implementation of needle‐tracking technology for real‐time transrectal ultrasound‐guided interstitial gynecological HDR brachytherapy: A feasibility study

**DOI:** 10.1002/acm2.70100

**Published:** 2025-05-08

**Authors:** Lindsey H. Bloom, Diandra Ayala‐Peacock, Rajesh Venkataraman, Brett Eckroate, Ryan Sanford, Junzo Chino, Yongbok Kim, Julie Raffi, Oana Craciunescu

**Affiliations:** ^1^ Department of Radiation Oncology Duke University Medical Center Durham North Carolina USA; ^2^ Eigen Health Services, LLC Grass Valley California USA; ^3^ Department of Radiation Oncology Rutgers University New Brunswick New Jersey USA

**Keywords:** brachytherapy, GYN, hybrid, image guidance, interstitial, ultrasound

## Abstract

**Purpose:**

To investigate the feasibility of adapting a commercial prostate biopsy system for transrectal ultrasound (TRUS)‐guided hybrid gynecological (GYN) high‐dose‐rate (HDR) brachytherapy (BT). Leveraging 3D‐TRUS and MR image fusion, the prototype system aims to improve real‐time needle placement accuracy.

**Materials and Methods:**

A second‐generation, multi‐imaging modality female pelvic phantom was developed to validate the system's feasibility. Software and hardware modifications, including user‐accessible calibration modules and a redesigned needle guide for multi‐needle insertion, were made to the pre‐existing commercial system to enable use for GYN BT applications. An end‐to‐end feasibility test was performed to acquire 3D‐TRUS images, perform contour‐based registration with pre‐implant MR, and insert six needles to targeted locations under real‐time TRUS guidance. A 30° tandem without ovoids was added to mimic a hybrid GYN implant. The most proximal and most distal distances between the planned needle track and the visible portion of each inserted needle were measured. A CT/MR image‐based treatment plan with a prescribed dose of 6 Gy was generated for the resulting 3D‐TRUS‐guided implant (tandem and needles) within the phantom.

**Results:**

The modified phantom improved needle visualization and insertion range by de‐gassing the silicone and increasing the window size. The system accuracy for average ± standard deviations from intended needle tracks was 1.31 ± 1.36 mm (proximal) and 2.04 ± 2.05 mm (distal). Post‐implant imaging confirmed needle placement and good target coverage. Needle placement was verified on CT/MR images and treatment plan quality was clinically acceptable.

**Conclusions:**

With enhanced needle placement accuracy and integrated clinical workflow, this study demonstrates the feasibility of adapting a commercially available prostate biopsy system for 3D‐TRUS‐guided hybrid GYN HDR BT.

## 1 INTRODUCTION

Treatment for locally advanced cervical cancer often involves the combination of radiation therapy delivered through external beam (EBRT) concurrent with chemotherapy (and possibly immunotherapy), followed by a high‐dose‐rate (HDR) brachytherapy (BT) boost.^1^ The addition of BT to a patient's treatment has been shown to significantly increase survival rates.^2^, ^3^

GYN HDR BT can be delivered using different techniques, including interstitial, intracavitary, or a combination (hybrid).^4^ Intracavitary applicators commonly used include tandem and ovoid (T&O) or tandem and ring (T&R).^4^, ^5^ In cases where the target volume is extensive (CTV_HR_ > 30 cc), interstitial implants are recommended in place of or in addition to intracavitary applicators in order to improve dose target coverage. Interstitial BT involves placing needles or catheters directly into the target area to provide comprehensive tumor coverage—a process heavily reliant on physician expertise.^4^, ^6^, ^7^, ^8^ For imaging, the use of CT and MR has been widely explored.^2^, ^8^, ^9^ Due to its easy accessibility and cost‐effectiveness,^10^ ultrasound (US) has become popular to guide tandem insertion^11^, ^12^, ^13^, ^14^ and has demonstrated potential for treatment planning.^14^, ^15^

One common clinical HDR workflow for complex GYN cases, as used at our institution, involves the use of multiple imaging modalities for both interstitial and intracavitary implant techniques. Pre‐implant MR imaging is acquired to assess disease extent following EBRT and determine the appropriate applicator for the BT implant. Applicator insertion is performed manually by the physician, usually under transabdominal or transrectal US guidance. When interstitial needles are used, the accuracy of the needle placement is assessed and adjusted using iterative CT acquisitions with an in‐suite CT. Post‐procedure CT and MR images are acquired for treatment planning, where the CT images are utilized for applicator reconstruction and source channel digitization, and the MR images are used for delineation of CTV_HR_ and OARs. Using applicators as landmarks, the CT and MR scans are rigidly co‐registered for treatment planning.

Incorporating real‐time US image guidance during needle insertion can enhance patient‐specific implant geometry and has the potential to mitigate the need for iterative imaging and applicator readjustment. Transrectal ultrasound (TRUS) offers visualization of relevant GYN anatomy that is comparable to MR.^16^, ^17^, ^18^ Although the use of real‐time TRUS guidance for catheter placement is well‐established in prostate biopsy^19^, ^20^ and prostate BT,^21^ it is less commonly used for GYN BT. Recent studies have demonstrated the feasibility of utilizing TRUS needle guidance for interstitial BT in cervical cancer treatments. Sharma et al. used 2D‐TRUS in conjunction with a perineal interstitial template,^18^ and Knoth et al. compared 3D‐TRUS and MR needle visualization with image‐guided adaptive BT.^22^ Both studies utilized intracavitary applicators with interstitial needles. This study investigates a novel method that utilizes 3D‐TRUS co‐registered with MR to provide interstitial needle guidance for hybrid GYN HDR‐BT.

One of the existing commercially available US‐based systems for prostate image‐guided biopsies is the Artemis system developed by Eigen Health Services (Grass Valley, California). Artemis is a 3D‐targeted US/MRI fusion‐based interventional needle guidance system that uses electro‐mechanical encoders to determine the location of the ultrasound probe at all times.^23^ Introducing intraoperative imaging, target planning, and needle guidance into the HDR workflow could mitigate the need for iterative imaging and enhance needle placement accuracy to improve target coverage and minimize normal tissue exposure. In a previous study, a female pelvic multi‐modality imaging phantom was designed and validated to use in conjunction with the Eigen Health needle‐tracking system.^24^ The purpose of this study is to investigate the feasibility of adapting the Artemis system, and its needle‐tracking technology, for clinical GYN HDR BT procedures. This study presents a refined end‐to‐end feasibility phantom test incorporating the Eigen needle‐tracking technology into a clinical HDR GYN BT workflow.

## 2 MATERIALS AND METHODS

### 2.1 Optimization of anatomical phantom construction

**FIGURE 1:**
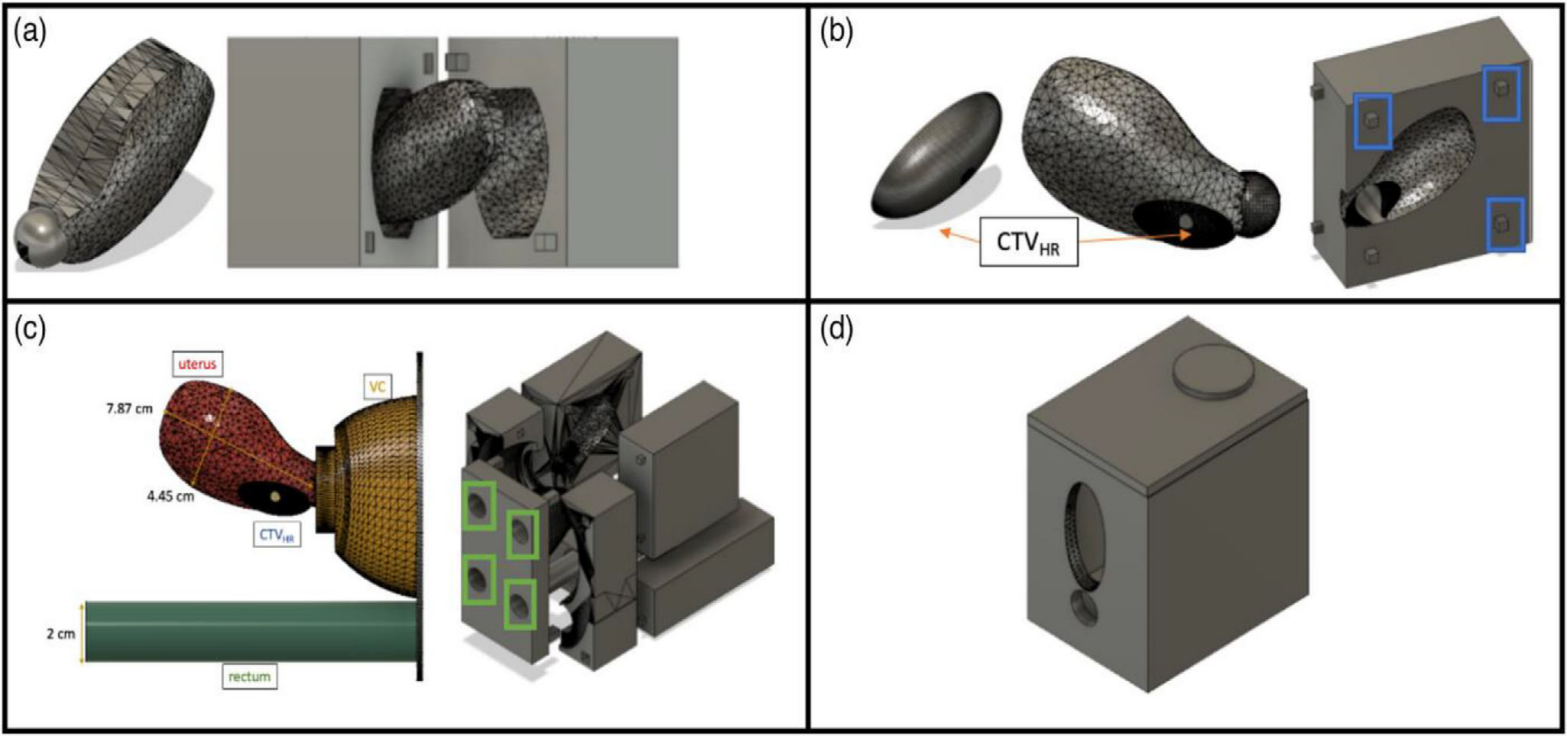
CAD models of previously validated first‐generation female pelvic phantom indicating design for: (a) uterus; (b) CTV_HR_ and uterus (with blue squares indicating pins to hold molds together); (c) vaginal canal, rectum, and uterus with relevant measurements (green squares indicate holes for pouring silicone during casting); (d) first‐generation box housing.^24^

In our previous study, a female pelvic phantom Figure 1 was constructed with silicone and agar materials and validated for use with the Eigen system.^24^ The phantom incorporated key anatomical structures including the rectum, uterus, vaginal canal, and an extra‐cervical CTV_HR_. Anatomical molds were designed in Fusion 360 (Autodesk Inc., California) and printed using Ultimaker S3 3D printers (UltiMaker, The Netherlands) with 5% in‐fill density polylactic acid (PLA). In this study, an optimized, second‐generation phantom was developed to address the limitations of the previous design and achieve improved visualization and integration with the needle guidance system.

To enhance anatomical visualization and eliminate air bubbles that caused shadowing artifacts during US imaging, a de‐gassing process was introduced. After the silicone was mixed, it was de‐gassed using a vacuum chamber (BACOENG, Minnesota) with a 110VAC pump for 10–15 min. Dragon Skin 20 (Smooth On Inc., Pennsylvania) was selected over the original MoldStar 20T (Smooth On Inc., Pennsylvania) silicone for its longer pot‐life, allowing sufficient time for air bubble removal before casting into anatomical molds. Dragon Skin 20 has properties comparable to Smooth‐On Mold Star 20T, which was previously selected for its ability to replicate the specific gravity and shore hardness of uterine soft tissue.^25^ To limit overall phantom construction time, Mold Star 20T was chosen to secure the CTV_HR_ and seal the silicone anatomy to the PLA housing box due to its shorter cure time.

The housing box of the phantom was modified to feature an increased window size, accommodating a wider range of needle insertion angles necessary for GYN HDR BT. This modification involved creating a square opening to facilitate lateral needle insertions without collision with the PLA housing box.

The phantom's background medium consisted of an agar formulation with cellulose powder as a thickening agent. This composition was chosen for its similar acoustic impedance to silicone, minimizing reflection and potential artifacts during ultrasound propagation.^24^, ^26^ After construction was complete, the phantom was refrigerated to set and preserve the agar. The phantom was removed from the refrigerator and allowed to reach room temperature prior to the experiment to mitigate temperature effects in US imaging. The silicone rectum was removed from the phantom and a cover filled with US gel was added around the TRUS probe to enable good contact between the probe and surrounding agar.

### 2.2 Eigen health prototype tracking system

**FIGURE 2:**
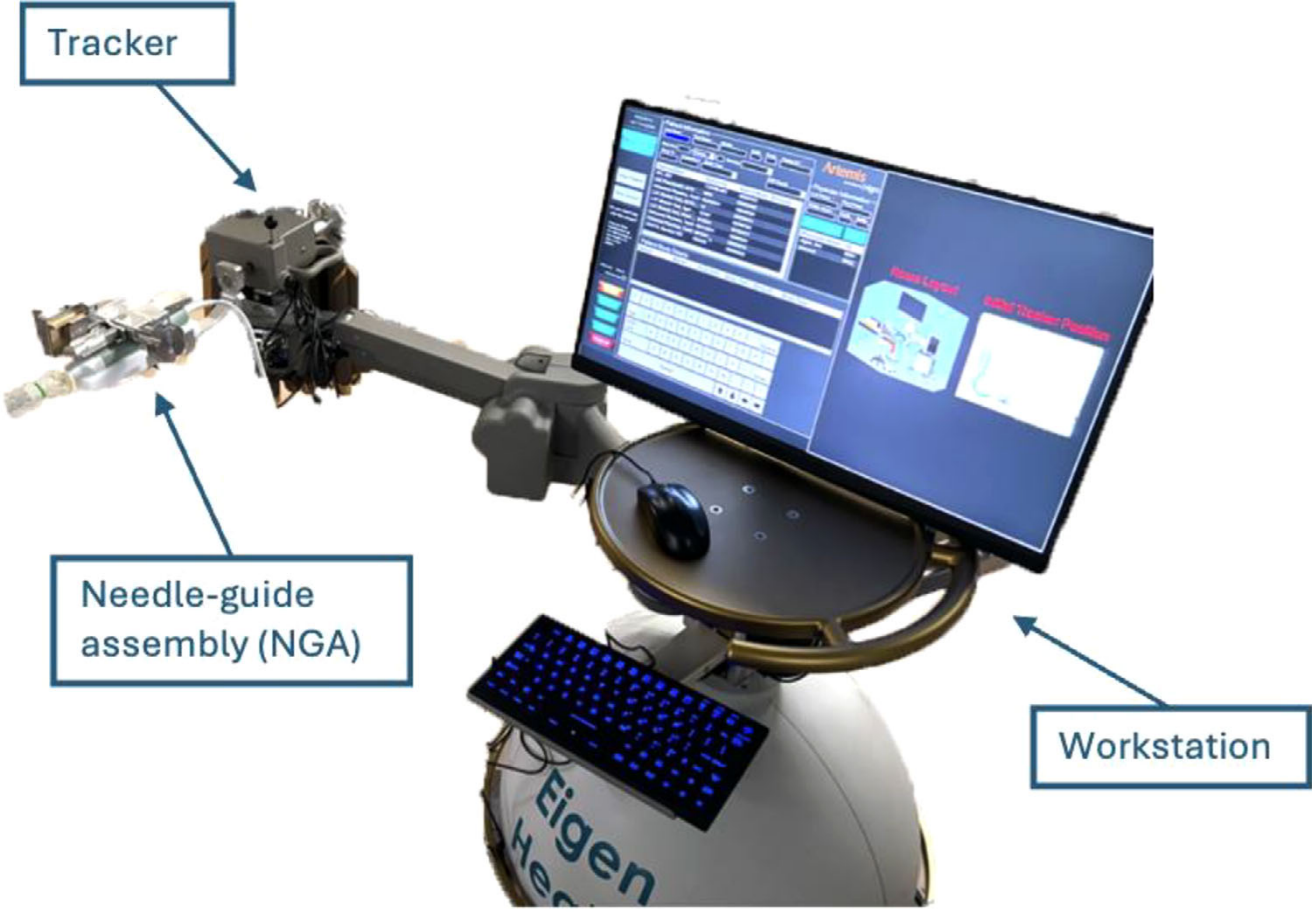
Eigen health services prototype system, featuring robotic arm with tracker, needle‐guide assembly, and mounted TRUS probe. TRUS, transrectal ultrasound.

The Eigen Health prototype tracking system Figure 2 consists of a manually assisted robotic arm that holds the TRUS probe, a needle guide assembly (NGA), a workstation that runs the software, and a keyboard and mouse to interact with the software. Altogether, the system guides the user in placing interstitial needles into the anatomy for precise targeting.

#### 2.2.1 System calibration

The system requires calibration of the tracker encoders (tracker calibration), NGA, and the captured US images. These calibrations are performed to ensure that the software knows where the probe is currently located and based on the calibrated values can prospectively show the needle guideline in the software where the interstitial needle will be placed. The sections below briefly describe what each different calibration step entails.

##### Tracker calibration

The system employs absolute electro‐mechanical encoders. To calibrate the encoders, the arm must be placed in a known position, defined as the “home position”, to set the baseline values for each encoder. The system uses two rotary encoders and one linear encoder. The rotary encoders are used for left/right probe rotation and for the needle path up/down angulation on the NGA. The linear encoder is used for capturing the advancement/retraction of the TRUS probe during the procedure.

##### US Calibration

The Eigen system is paired with the US system using a frame grabber, which captures the US frames. With this, the system can track the US depth and acquire images for performing 3D‐TRUS reconstruction. The US calibration is performed by capturing specific templates based on the current depth to define the resolution of the acquired frame. Other parameters that are captured are left/right marker, resolution, grayscale region, US system specification, and US probe model. For this study, we used our clinical BK Specto ultrasound system with an E14CL4b transrectal transducer (BK Medical, Denmark).

##### Needle‐guide assembly (NGA) calibration

The final calibration is the NGA calibration. The NGA moves the needle path in two distinct ways. One is by rotating the needle guide to access the posterior or anterior of the cervix, and the second is by moving it up or down vertically. Due to manufacturing and assembly tolerances, the NGA kinematic modeling could be significantly different; the kinematic parameters of the NGA are calibrated by inserting a needle and then calculating the difference between the predicted path and the actual needle location. This was performed using multiple insertions with different NGA positions to ensure we accounted for needle deflection and phantom irregularities.

#### 2.2.2 Needle‐guide design

The original needle guide (Eigen Health, California, 590‐01002‐00^27^) that mounts on the NGA was designed for prostate biopsy procedures. The needle guide was adapted and redesigned within OnShape CAD software (PTC, Massachusetts) to facilitate the release of the needle post‐insertion and allow for the insertion of multiple interstitial needles, as is typically required for HDR GYN BT procedures. The redesigned needle guide was printed using Formlabs Form 3B printers (Formlabs, Massachusetts) with BioMed Clear resin (Formlabs, Massachusetts), chosen for its sterilizability, biocompatibility, and rigidity.^28^

### 2.3 End‐to‐end feasibility test

**FIGURE 3:**
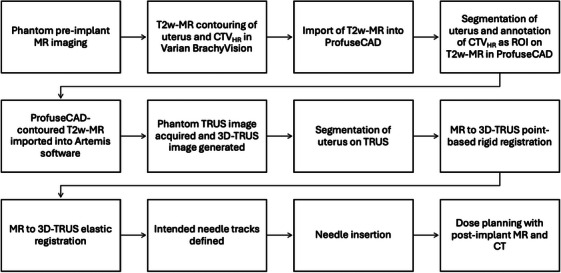
Flow chart of end‐to‐end feasibility test components.

Following the construction of the phantom (See Section 2.1) and re‐design of the needle guide (See Section 2.2.2), an end‐to‐end feasibility test Figure 3 was performed to evaluate the TRUS‐guided needle insertion workflow. The main components of this test are detailed below.

#### 2.3.1 Pre‐implant CT and/or MRI imaging

Pre‐implant imaging was performed to assess phantom quality and to define contours for needle guidance. For this experiment, a Siemens Skyra 3T MR (Siemens Healthineers, Germany) scanner was used following our institutional‐specific GYN MR imaging protocol body coil: T1w—3d SPGR 1mm isotropic with TR = 3.7 ms and TE = 1.3 ms, FOV 300 × 300 mm and T2w—axial with TR = 2000 ms and TE = 121 ms, FOV 300 × 300 mm. Using BrachyVision (Varian Medical Systems, California), all components of the anatomical phantom, including the uterus, cervix + extracervical CTV_HR_, vaginal canal, and rectum, were contoured.

The T2w‐MR images were imported into ProfuseCAD, Eigen's AI‐powered fusion‐enhancement contouring software, where the uterus and CTV_HR_ were re‐contoured to be exported in the proprietary format accepted by the Eigen needle tracking software, Artemis. Within ProfuseCAD, the uterus was segmented and the cervix + extracervical CTV_HR_ was annotated as a region of interest (ROI). The T2‐w MR images, with the associated ProFuse contours for the uterus and CTV_HR_, were imported into the Artemis software for use during the experiment.

#### 2.3.2 TRUS image acquisition

**FIGURE 4:**
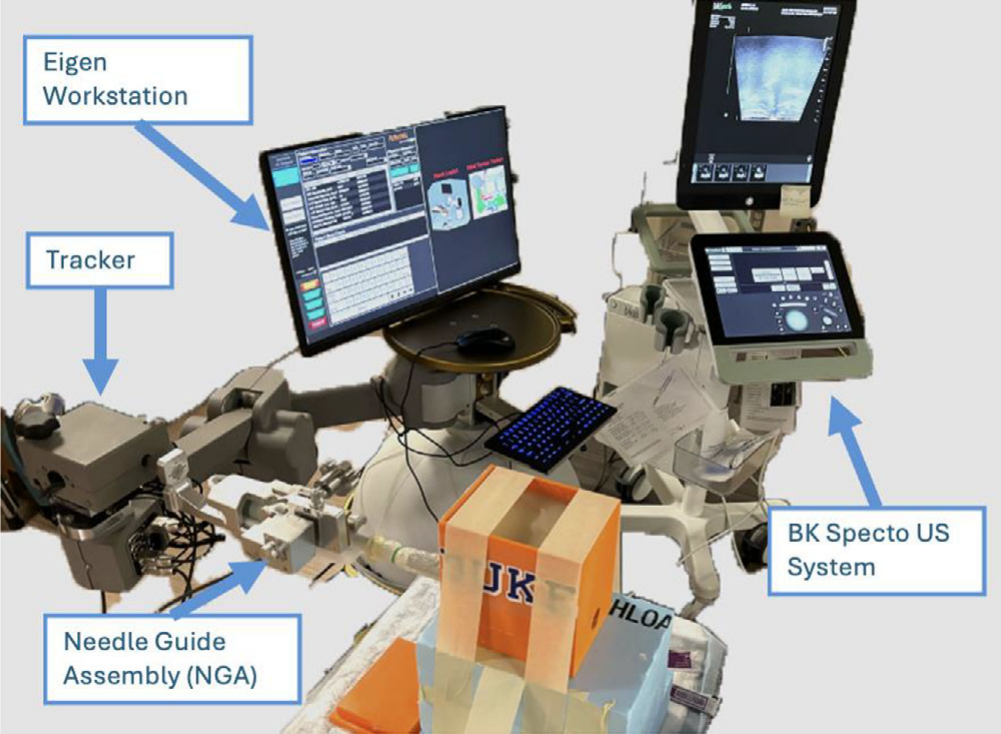
Experimental set‐up showing the integration of the BK Specto US system, the Eigen prototype guidance system and its components, and the female pelvic phantom.

The experimental setup Figure 4 included mounting the TRUS probe onto the stepper cradle on the prototype device's robotic arm. The E14CL4b TRUS probe was inserted into the phantom's rectal opening to the point of visualizing the maximum extent of the uterus. A 170° lateral‐to‐lateral sweep was performed using the BK Specto US system at 9 MHz frequency to enhance visibility.^29^ Within Artemis, the acquired images were used to generate a 3D‐TRUS volume of the phantom.

#### 2.3.3 Uterine contouring on TRUS

The uterus was segmented in the transverse and sagittal planes of the 3D‐TRUS, followed by manual refinements to match the uterine wall's exterior boundary.

#### 2.3.4 MR/TRUS contour‐based registration

A landmark‐based rigid registration was first performed by marking two corresponding uterine contour points between T2‐w MR (pre‐procedure, as contoured in ProFuse) and TRUS (intra‐procedure) in the sagittal and transverse planes, sequentially. Using the defined uterine contours on T2‐w MR and TRUS, a deformable registration (surface‐based elastic registration) based on Shen^30^ was performed, resulting in the CTV_HR_ defined on the 3D‐TRUS volume. Visual verification confirmed the registration.

#### 2.3.5 Applicator insertion

The guidance system, initially designed for single‐point prostate biopsies, was adapted to support needle‐path‐based planning essential for hybrid GYN HDR BT source placement. Eigen provides a prostate LDR module that can be added to the system to visualize the intended needle targets as “rods” compared to points. Using this LDR module, five interstitial 17‐gauge titanium needles (Varian Medical Systems, California, GM11009730) and one interstitial 17‐gauge stainless steel needle (Varian Medical Systems, California, GM11009520) were inserted. The module requires a minimum needle spacing of 5 mm to prevent collisions. Note that we no longer use stainless steel needles clinically, but one was included in this experiment since it was mixed in with our needles set aside for non‐clinical use. Following interstitial needle insertions, an MR conditional titanium 30° 6 cm tandem applicator (Varian Medical Systems, California, AL07522001) was inserted to demonstrate the compatibility of the guidance system with intracavitary applicators. Due to phantom limitations, the tandem was inserted without ovoids. Results were assessed by comparing the measured distance between the intended needle track and the actual inserted needle. While the commercial prostate biopsy system has demonstrated a point‐based targeting accuracy of 1.2 ± 1.1 mm,^31^ a deviation tolerance of up to 5 mm was used in this experiment, per physician recommendations for clinical use.

#### 2.3.6 Post‐implant imaging and treatment planning

Post‐implant, a fan beam AiroCT (Stryker Inc., Michigan) scan was taken to capture the final implanted positions of the interstitial needles and tandem. A CT series was acquired with all needles left in place with coded markers in each needle. This CT dataset was used to digitize the interstitial needles and tandem channels for treatment planning. Post‐implant MR, following the same protocol as pre‐implant imaging, was performed for the delineation of target and OARs. Prior to the MR scanning, the stainless‐steel needle was removed. The post‐implant CT images and MR were rigidly co‐registered, based on applicators and phantom anatomy. A treatment plan was generated in the BrachyVision (Varian Medical Systems, California) treatment planning system (TPS). The treatment plan quality was evaluated based on dosimetric clinical goals for a 6 Gy treatment fraction.

## 3 RESULTS

### 3.1 Optimization of anatomical phantom construction

**FIGURE 5:**
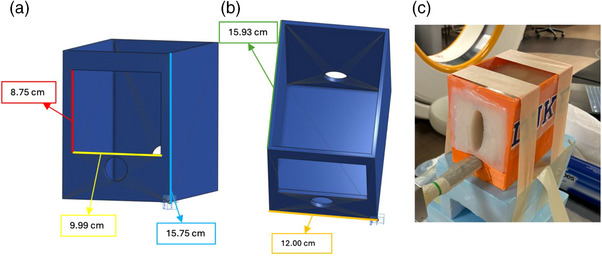
(a,b) CAD model of redesigned box housing with window opening with relevant measurements; (c) fully constructed phantom with silicone anatomy inserted and silicone rectum removed for probe insertion.

**FIGURE 6:**
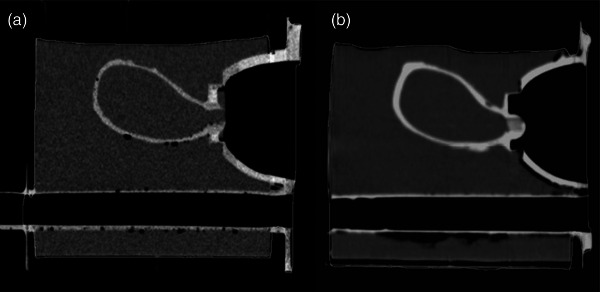
Sagittal CT of (a) first‐generation phantom following original construction method and (b) second‐generation phantom with degassing process incorporated.

The box housing dimensions were 15.93 × 12.00 × 15.75 cm (L × W × H), with a window opening of 9.99 × 8.75 cm (W × H) Figure 5. To prevent agar leakage, the face of the box was coated with Mold Star 20T silicone. The redesigned window allowed for increased lateral rotation of the TRUS probe and attached needle guide for needle insertion without collision with the box housing. The extended pot‐life of Dragon Skin 20 silicone facilitated the complete removal of air bubbles during the degassing process, enhancing anatomical visualization Figure 6.

### 3.2 New needle guide‐design

**FIGURE 7:**
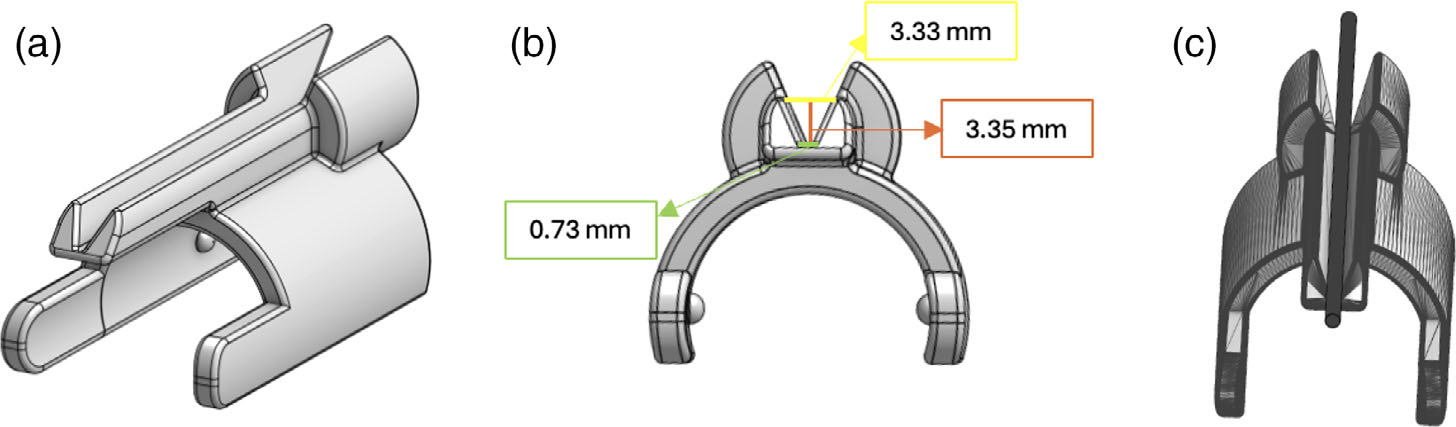
(a) CAD model of needle‐guide displaying new dovetail design with (b) measurements; (c) model of needle‐guide with a rod of 17‐gauge diameter, mimicking needle compatibility.

The open‐top needle guide design Figure 7a,b featured a dovetail with a depth of 3.35 mm. The dovetail track began at a width of 3.33 mm and descended at an angle of 51.83° to a width of 0.73 mm, suitable for a 17‐gauge (1.43 mm) needle to be placed and guided Figure 7c. This design permitted easy release of the needle to facilitate subsequent needle insertions. The encoders were calibrated with the redesigned needle guide mounted.

### 3.3 End‐to‐end feasibility test

#### 3.3.1 Pre‐implant imaging

**FIGURE 8:**
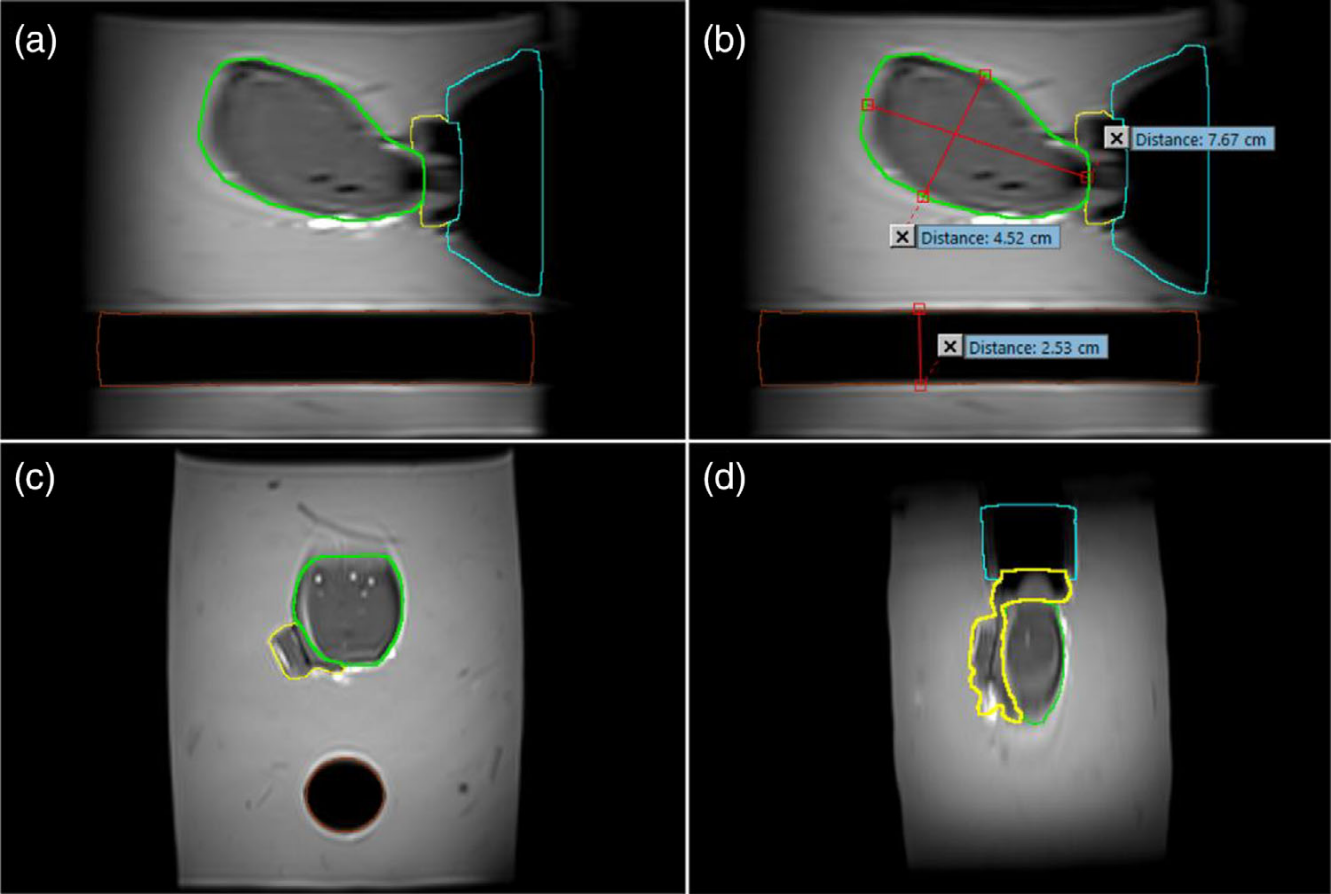
(a) Pre‐implant sagittal T2w‐MR image showing uterus (green), cervix + extracervical CTV_HR_ (yellow), vaginal canal (cyan), and rectum (red); (b) Measurements of uterus and rectum on BrachyVision TPS; (c) transverse T2w MR; (d) coronal T2w‐MR.

Based on the BrachyVision contours of the T2w‐MR pre‐insertion images Figure 8, the contoured uterine volume was 80.05 cc, with a cervix to fundus length of 7.67 cm and widest height of 4.52 cm. The cervix + extra‐cervical CTV_HR_ had a volume of 23.00 cc. The diameter of the rectal cavity after removal of the silicone rectum was 2.53 cm.

After contouring the T2‐w MR within ProFuse, the uterine volume imported into Artemis for subsequent steps was 75.82 cc and the manufactured CTV_HR_ was 27.53 cc.

#### 3.3.2 TRUS image acquisition

**FIGURE 9:**
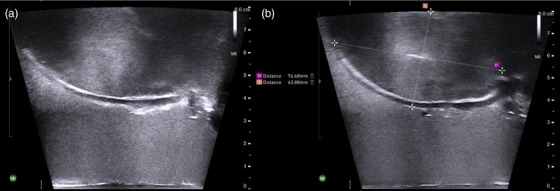
(a) Sagittal TRUS of the uterus with (b) displaying the measurements (in mm) of the uterus, as measured on the BK Specto US system. TRUS, transrectal ultrasound.

The phantom was imaged with TRUS with a depth of 8.0 cm. The uterus is shown in Figure 9, with dimensions closely matching those of the pre‐implant MR: a cervix to fundus length of 7.67 cm and widest visualized height of 4.39 cm.

#### 3.3.3 Uterine contouring on TRUS

**FIGURE 10:**
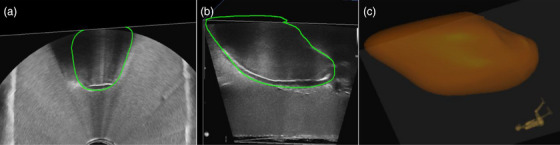
(a,b) TRUS contour of the uterus (green) displayed in the transverse (a) and sagittal (b) planes; (c) resulting in 3D‐rendered uterine volume. TRUS, transrectal ultrasound.

The uterine segmentations on the real‐time TRUS images are shown Figure 10a,b with the resulting 3D‐rendered uterine volume Figure 10c. The TRUS uterine volume generated on the needle‐tracking software was 76.4 cc.

#### 3.3.4 MR/TRUS contour‐based registration

**FIGURE 11:**
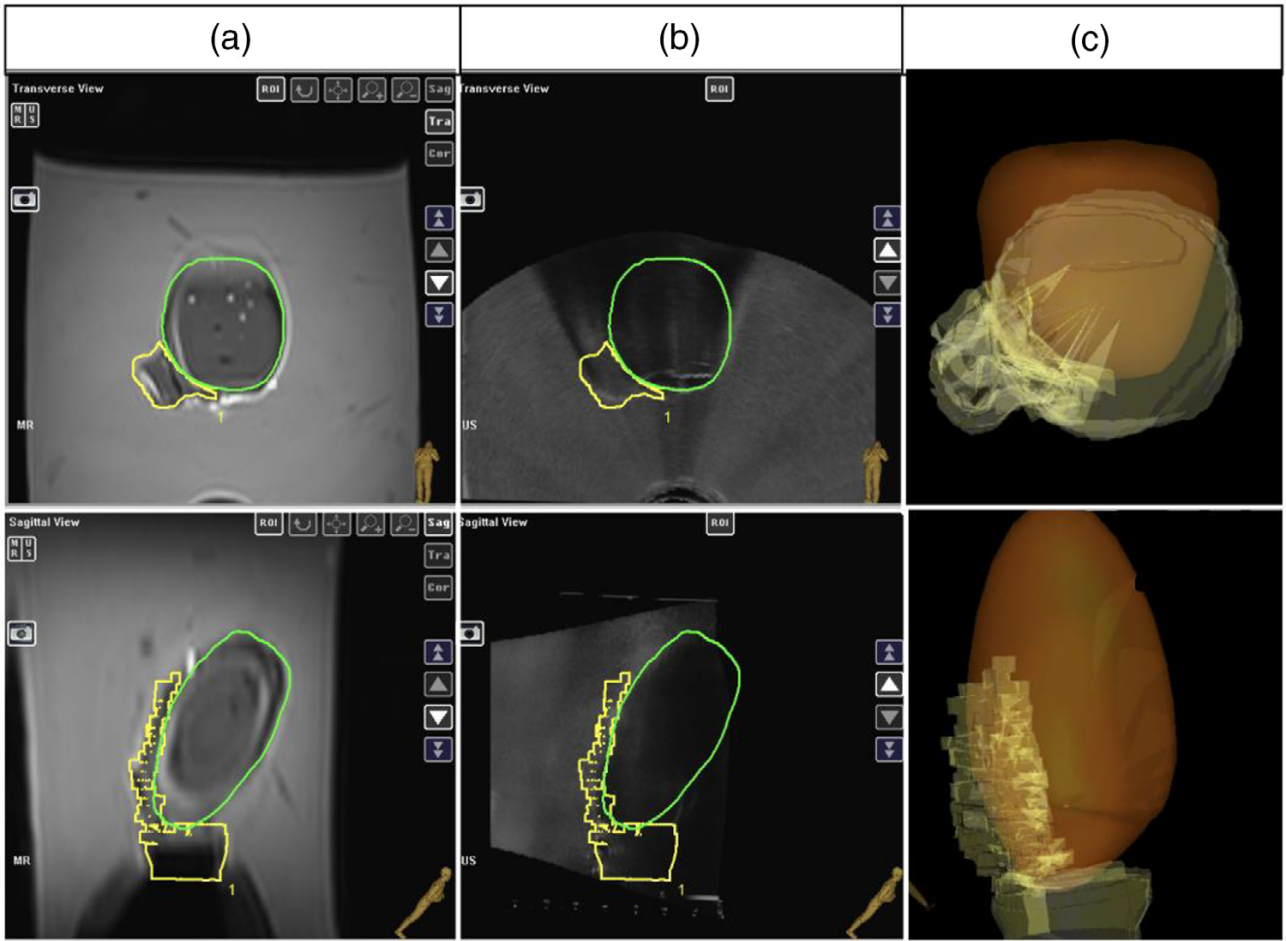
Transverse (top) and sagittal (bottom) views of contours used for targeting after T2w‐MR to TRUS registration. Post‐registration uterine (green) and CTV_HR_ (yellow) contour on MR (a) and TRUS (b); (c) 3D‐rendered model of the uterus (orange) and CTV_HR_ (yellow) for needle‐placement and targeting. TRUS, transrectal ultrasound.

Following the point‐based rigid registration and surface‐based elastic registration algorithm, the T2w‐MR was deformed to match the real‐time TRUS, and the contours used for needle targeting were generated Figure 11.

#### 3.3.5 Applicator insertion

**FIGURE 12:**
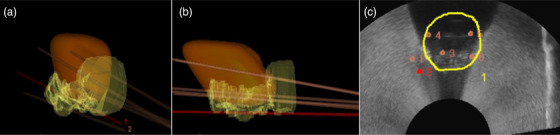
(a,b) 3D‐rendered uterus (orange) and CTV_HR_ (yellow) showing six intended needle tracks; (c) transverse view of the six needle insertion points in the plane with the cervix (yellow). Red “2” indicates the needle track that the probe was in‐plane with at the time of screen capture. Yellow “1” indicates the annotated cervix, denoted as part of the defined ROI. ROI, region of interest.

**FIGURE 13:**
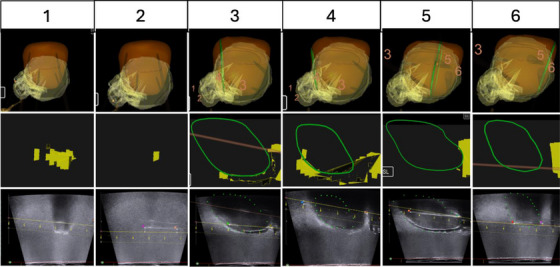
Top) 3D‐rendered uterus (orange) and CTV_HR_ (yellow) with needle target locations indicated; Middle) the contour of CTV_HR_ (solid yellow) and uterus (green) in the plane of the TRUS transducer when rotated to the target point of interest; Bottom) live TRUS image with the intended needle path (solid yellow) displayed in the same plane as the actual needle insertion. TRUS, transrectal ultrasound.

The intended needle paths chosen for guided insertion using the LDR module spanned the uterus and CTV_HR_ Figure 12. Figure 13 demonstrates the targeting process of each needle across the 3D‐rendered targeting volumes, the contours in‐plane with the intended needle path, and the TRUS image in that plane. The anterior‐posterior distance between the intended needle track was measured at both the most distal end of the needle (needle tip) and the most proximal point of needle visualization to account for deviation along the depth of the needle. The measurements between the intended needle path and the actual needle path are shown in Table 1. The average deviation across all paths for the proximal measurements was 1.31 ± 1.36 mm and 2.04 ± 2.05 mm for the needle tip measurements. The stainless steel needle is indicated in the third column of Figure 13 and Table 1.

**TABLE 1:** Measured distance between the intended line of insertion and actual needle insertion for six intended needle paths.

Target	1	2	3	4	5	6	Avg
Proximal distance from the intended track (mm)	0	3.67	0.41	1.49	0.41	1.9	1.31 ± 1.36
Distal distance from the intended track (mm)	0	4.48	0.27	2.99	4.07	0.41	2.04 ± 2.05

#### 3.3.6 Post‐implant imaging and treatment planning

**FIGURE 14:**
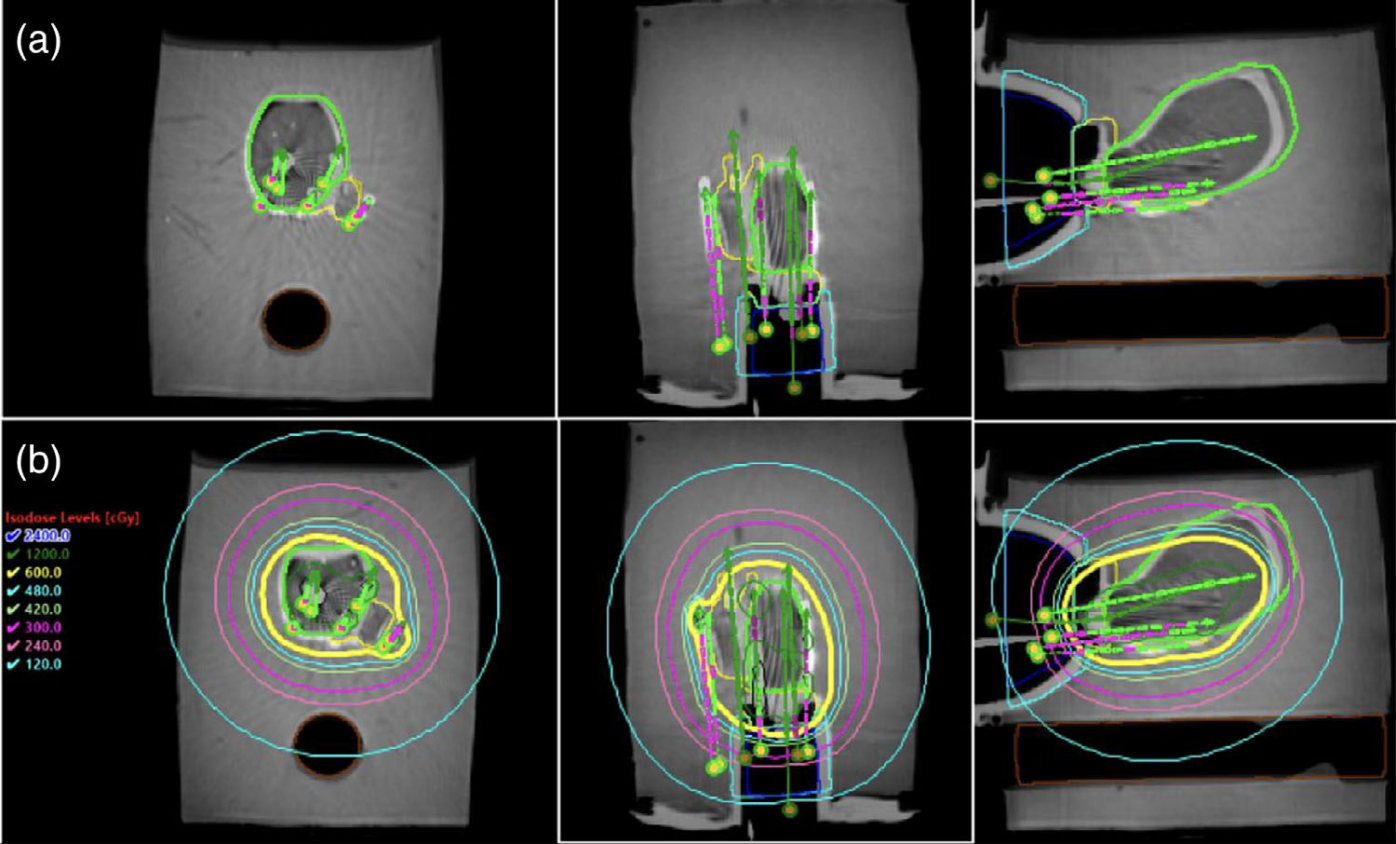
(a) post‐implant CT and T2w‐MR registration displaying uterus (green) and CTV_HR_ (yellow) contours with digitized applicators (green+ purple); (b) dose plan overlay showing isodose levels superimposed on the registered image.

The feasibility of creating a clinical‐like plan is shown in Figure 14. Table 2 summarizes the dosimetric endpoints for this test plan.

**TABLE 2:** Target coverage and OAR‐sparing clinical goals with the prescribed dose of 6 Gy.

Rx dose			6 Gy
Clinical goals			Actual dose
CTV_HR_	Priority 1	D_90_ ≥ 6 Gy	6.78 cGy
	Priority 1	D_98_ ≥ 4 Gy	5.66 cGy
Rectum	Priority 2	D 2.0 cm^3^ ≤ 3.9 Gy	1.74 cGy
Vagina	Priority 3	D 2.0 cm^3^ ≤ 6.8 Gy	6.40 cGy

## 4 DISCUSSION

The aim of this study was to explore the feasibility of adapting a commercially available prostate biopsy system for TRUS‐guided GYN HDR BT use. Through multiple female pelvic phantom experiments, we have demonstrated the capability of needle‐tracking under TRUS guidance and consistently shown reproducible and reliable outcomes.^32^, ^33^, ^34^

A previously designed multi‐modality female pelvic phantom was improved by including a de‐gassing process and design modifications. The de‐gassing step effectively eliminated air bubbles from the silicone anatomy, enhancing TRUS visualization and image quality. To accommodate this process, we selected an alternative medical‐grade silicone, commonly used in prosthetics. While this material allowed time for de‐gassing prior to curing, its increased viscosity presented challenges during phantom construction, occasionally preventing the complete filling of narrow mold spaces and causing non‐uniformities and breakages. In such cases, remaking and curing the affected regions extended the overall production time. To improve the needle insertion range, the box housing window was modified and enlarged, allowing lateral needle insertions without interference from the box housing material.

Several software and hardware design changes were necessary to adapt the system for GYN HDR BT use, including needle guide modifications to allow for multiple needle insertions and needle‐path‐based insertion guidance for subsequent HDR planning. Prior to our collaboration, calibration information embedded within the system was inaccessible to end users. Following our feedback, user‐friendly calibration modules were added to the system for physics acceptance and commissioning in Radiation Oncology environments.

Initially, the system did not support the insertion of multiple needles, which is necessary for GYN BT clinical applications. To address this, we developed a needle guide that allows successive insertion of multiple needles to replicate a clinical implant and allows treatment planning with all needles in position.

Prior unpublished data revealed that both agar temperature during TRUS imaging and the presence of the silicone rectum affected image scaling. At colder temperatures, agar slows the speed of sound. Additionally, the speed of sound is significantly slower when propagating through silicone than through water or tissue,^35^ resulting in a lateral stretching effect. By removing the rectal wall and allowing the phantom to reach room temperature before TRUS imaging, these effects were mitigated. Moreover, the removal of the silicone rectum also improved contact and consistency in US propagation by eliminating visibility inconsistencies. However, in this study, the TRUS uterine segmentation volume remained larger than that of the MR. Thus, work is being done to further explore the discrepancies, but this limitation did not affect the end point of our study.

The feasibility of using the Eigen prototype system in GYN HDR BT procedures was assessed through a pelvic phantom study simulating clinical workflow. Our results consistently achieved the physician‐recommended target deviation of 2–5 mm for clinical procedures. Following needle insertion, we completed the comprehensive clinical workflow, including post‐implant imaging and realistic treatment planning, achieving optimal dose target coverage and OAR sparing. The clinical relevance of the calculated plan was limited due to the lack of bladder as well as the large spacing between the rectum and uterus in the phantom design. Although ovoids were omitted from the T&O applicator due to phantom limitations, the system demonstrated compatibility with interstitial needles and intracavitary tandem applicators commonly used in GYN HDR hybrid treatments. The results of this study support the feasibility of incorporating this system for GYN HDR treatments.

**FIGURE 15:**
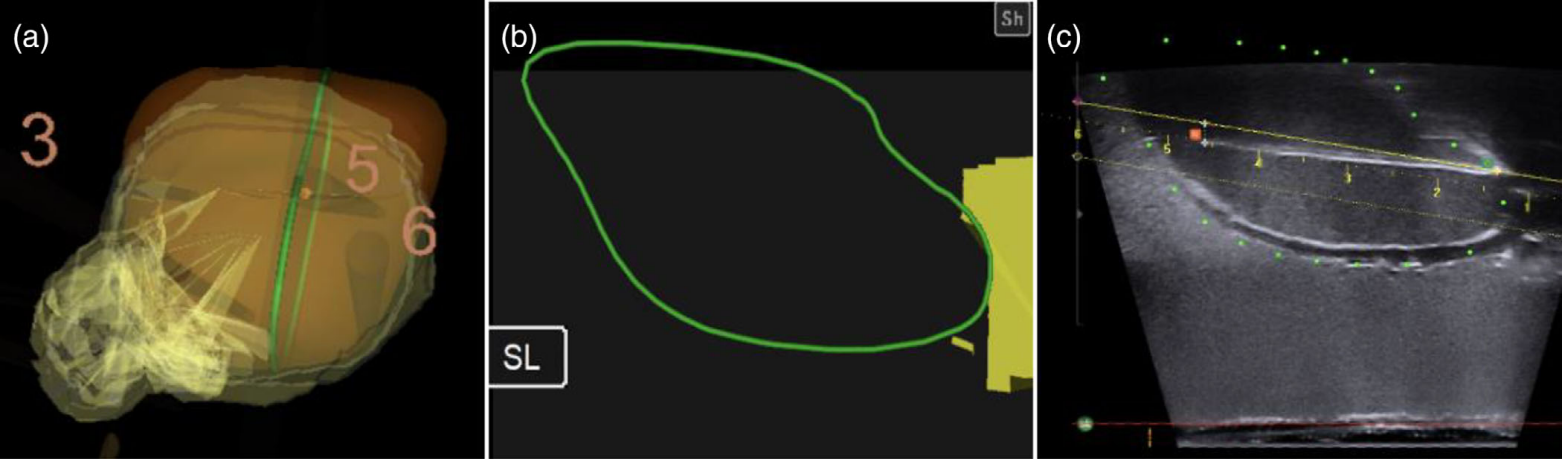
Enlarged view of targeting with previously bent needle for point 5. (a) 3D‐rendered uterus (orange) and CTV_HR_ (yellow) targeting point 5, with points 3 and 6 also in view; (b) contour of CTV_HR_ (solid yellow) and uterus (green) in the plane of the TRUS transducer when rotated to the plane of actual needle insertion. No intended needle track is shown, as the intended target was in a different plane; (c) live TRUS image in the plane of the TRUS transducer when rotated to the plane of actual needle insertion with points used for deviation measurement from the intended needle path (solid yellow) shown. TRUS, transrectal ultrasound.

While our studies have shown promise for the implementation of needle‐tracking software into GYN BT, several limitations remain in our approach. First, the only needles that were accessible for use in our studies had been previously used and therefore were no longer perfectly straight. Our study focused on the anterior‐posterior deviation from the intended target line; however, there were also lateral deviations for some needles that could not be accurately measured. For needles that deviated out of a plane with the transducer Figure 15, the TRUS probe was rotated to visualize the needle and measure anterior to posterior offset from a planned position with the intended needle track superimposed. The current system design only allows for visualization of needle insertion in the sagittal plane. This is due to the importance of viewing the needle entry and pathway where dwell positions will be defined during planning. The degree of rotation and distance to the insertion plane were not quantified. We also did not consistently measure the depth to which each needle was inserted, as this was not one of the endpoints of this feasibility study. In the case that the needle was bent in the anterior‐posterior direction, the greater insertion depth into the phantom would lead to a greater deviation.

The precision of needle guidance from the prototype system relies on accurate registration between the MR and TRUS images. The proprietary surface‐based elastic registration algorithm, based on a 3D brain structures study,^30^ embedded in the system estimates a deformation field between the uterine contours to deform the pre‐insertion MR image to the real‐time TRUS. Our group is currently working on validating the deformable registration algorithm for GYN applications, and preliminary findings were reported.^32^, ^36^

Secondly, the female pelvic phantom can be improved further to enable full TRUS visualization. In its current form, the entirety of the uterus cannot be fully visualized within the field‐of‐view of the BK Specto maximum 8.0 cm depth. This inhibits imaging of the uterine fundus which could lead to possible errors in TRUS uterus segmentation and volume discrepancies. While the current phantom also does not have a bladder, which would be useful for treatment planning studies, ongoing work aims to redesign the phantom for full anatomical visualization. The preliminary data served as the basis for an approved IRB patient study at our institution using the prototype system to acquire 3D TRUS images for patients undergoing gynecologic BT. This patient study will help explore the real‐world variation in uterine‐cervix anatomy and visibility with TRUS.

Lastly, there are several limitations in the Eigen system workflow that can be addressed to enable smoother integration into clinical GYN BT use. Currently, users must use Eigen's ProfuseCAD segmenting software to re‐contour the uterus and CTV_HR_ and generate the proprietary format required for Artemis. The replication of contours across different software introduces the possibility for variability in segmentation results. The uterine volume discrepancies across software can negatively affect the reliability of the surface‐based elastic registration results and, ultimately, the targeting accuracy. Also, as observed in Figure 9, the annotation of the CTV_HR_ from ProfuseCAD is not interpolated across slices. We are working to address these limitations by enabling direct Artemis‐compatible imports from clinical DICOM RTSTRUCT files. Additionally, the system's exports contain private tags, requiring conversion to DICOM‐RT format for compatibility with clinical TPSs such as BrachyVision. We have developed a MATLAB (MathWorks, Massachusetts) code to process the 3D TRUS files for import into Eclipse.^37^ Another limitation is the inability to further analyze or process previous scans following the conclusion of the procedure. Work is ongoing to incorporate a Physics QA module into the system for thorough acceptance testing and commissioning, as required in clinical environments. These findings all contribute to the collaborative effort to create a modified, validated system that is dedicated to GYN procedures.

## 5 CONCLUSION

Integrating needle‐tracking into GYN HDR hybrid BT treatments promises enhanced interstitial needle placement, reducing the need for re‐imaging, re‐insertion, and over‐implanting with additional needles to ensure optimal dosimetry. This study demonstrated the feasibility of adapting a commercial prostate biopsy system for use in TRUS‐guided GYN HDR BT. Through a comprehensive phantom study, the capability of prospective, non‐iterative targeted TRUS‐guided needle insertion was established, with substantial software and hardware modifications implemented to support GYN HDR BT procedures. The end‐to‐end feasibility test yielded promising results in integrating the Eigen Artemis needle‐tracking system into a clinical HDR GYN protocol, though additional system adjustments are necessary for practical HDR application. In conclusion, this work contributes to the advancement of TRUS‐guided GYN HDR BT and lays the groundwork for future research and development in this critical area of medical physics.

## AUTHOR CONTRIBUTION

*Lindsey H. Bloom*‐wrote the entirety of the manuscript, conducted all experiments to gather data, formed conceptualization, created phantom and modifications. *Diandra Ayala‐Peacock*‐participated in clinical data acquisition, provided critical revisions of the manuscript, and project supervision. *Rajesh Venkataraman*‐developer of software used in the manuscript provided software validation, data collection, provided manuscript revisions, and project supervision. *Brett Eckroate*‐phantom creation and manuscript revision. *Ryan Sanford*‐assisted with data collection, phantom creation, and manuscript revision. *Junzo Chino*‐contributed to clinical guidance and manuscript revision. *Yongbok Kim*‐supervised the technical aspects of the study and contributed to the writing and editing of the manuscript. *Oana Craciunescu* and *Julie Raffi*‐ provided senior author guidance, overall project oversight, and critical manuscript review.

## CONFLICT OF INTEREST STATEMENT

This research was partially supported by a grant of $3000 from Eigen Health (Grass Valley, CA) to assist with student research funding.

## COALITION STATEMENT

Yes, all co‐authors have reviewed this journal's licensing options and, on behalf of all co‐authors, I confirm that all co‐authors have the full power, authority, and capability (i) to agree to the terms of one of the licenses offered by this journal and (ii) to grant the rights set forth in such license for the publication of this submission. The submitting author is expected to consult all authors to find out whether any of their funders have a policy that restricts which kinds of license they can sign, for example, if the funder is a member of Coalition S. Information about licensing options is available here.

## PREVIOUS ONLINE POSTING

Preliminary work/data presented at AAPM 2023 (Houston Texas): Bloom L RJ, Venkataraman R, Eckroate B, Kim Y, Chino J, Ayala‐Peacock D, Craciunescu O. Implementation of Existing Needle Tracking Technology for Real Time Transrectal (TRUS)‐guided Interstitial Gynecological HDR Brachytherapy: A Feasibility Study. presented at: AAPM Annual Meeting; 2023; Houston, Texas. and ASTRO 2023 (California): Ayala‐Peacock D RJ, Bloom L, Venkataraman R, Chino J, Eckroate E, Craciunescu O. REAL‐TIME PRECISION NEEDLE TRACKING FOR GYNECOLOGICAL INTERSTITIAL BRACHYTHERAPY: A Phantom Feasibility Study. presented at: ASTRO; 2023; California. Preliminary work/data also published in MS thesis: Bloom L RJ, Ayala‐Peacock D, Venkataraman R, Craciunescu O. Developing Needle‐Tracking Techniques with Real‐Time Transrectal Ultrasound Guidance in GYN Hybrid HDR Brachytherapy. Duke University; 2024. References to all of the above are included in manuscript.

## Data Availability

Data is available as supplemental files associated with the online journal article.
